# Facing COVID-19 Between Sensory and Psychoemotional Stress, and Instrumental Deprivation: A Qualitative Study of Unmanageable Critical Incidents With Doctors and Nurses in Two Hospitals in Northern Italy

**DOI:** 10.3389/fpsyg.2021.622894

**Published:** 2021-04-12

**Authors:** Ines Testoni, Chiara Franco, Enrica Gallo Stampino, Erika Iacona, Robert Crupi, Claudio Pagano

**Affiliations:** ^1^Department of Philosophy, Sociology, Education and Applied Psychology (FISPPA), University of Padova, Padua, Italy; ^2^Emili Sagol Creative Arts Therapies Research Center, Faculty of Social Welfare and Health Sciences, University of Haifa, Haifa, Israel; ^3^NewYork-Presbyterian Queens Hospital, New York, NY, United States; ^4^Clinica Medica 3, Department of Medicine - DIMED, University of Padova, Padova, Italy

**Keywords:** COVID-19 pandemic, critical incidents, psychoemotional stress, death, dehumanisation

## Abstract

**Background:** The COVID-19 pandemic severely strained the already unprepared Italian healthcare system. This had repercussions on healthcare workers, stemming, in particular, from a lack of clear guidelines, adequate protective equipment, and professional preparedness. Such conditions were especially prevalent in Northern Italy.

**Objectives:** This study aimed to examine COVID-19-related professional and psychoemotional stress among nurses and doctors in two hospitals in Northern Italy, along with the worst critical incidents affecting healthcare personnel. A parallel objective was to elicit healthcare professionals' opinions about what changes are needed in the healthcare system's operations, as well as about the relational/emotional skills that are needed to better manage relationships with patients in emergency situations.

**Participants:** Snowball sampling was used to recruit participants and yielded 17 hospital professionals: six nurses (five female and one male) and 11 doctors (seven male and four female). Three of these professionals worked in intensive care and the others in different wards. All had close contact with COVID-19 patients.

**Methods:** The study employed a qualitative research design, using in-depth interviews of ~60 min each that were conducted *via* Skype video calls. The interviews were recorded and transcribed, then analysed. The qualitative analysis employed mixed methods to identify the most relevant and recursive themes from the interviews.

**Results:** Four fundamental themes emerged from our analysis of the interview texts: (1) disorganisation and psychoemotional stress; (2) urgency and critical incidents; (3) everything surreal; and (4) disruptions in empathetic relationships with patients.

**Conclusions:** Through our analysis of the interview narratives, we found that systematic and in-depth psychological training is needed to prepare professionals for (1) altered relationships with patients in emergencies; (2) use of exceptional medical equipment; (3) elaboration of new bioethical models suitable for disasters and pandemics; and (4) engagement with the themes of death and dying.

## Introduction

The COVID-19 pandemic has tested the Italian healthcare system and significantly affected the relationships among healthcare professionals, patients and their families. The pandemic occurred during a time of critical transformation during which healthcare professionals were already committed to changing their model for interacting with patients. In fact, the doctor/nurse-patient relationship had been evolving over the past two decades, corresponding with shifts in approaches to illness and health. The most significant of these changes has been a movement away from understanding disease as a compromise of biological structures and functions—on which doctors were the unique authority—to a new perspective that views health as a complex phenomenon with intertwined biological, psychological, social, and spiritual aspects. The latter view requires a different relationship between healthcare professionals and patients [i.e., one that moves away from authoritative frameworks and towards more humanistic ones, such as those recommended by the medical humanities and patient-centric approaches (Pirone, 2018)]. Despite a continuously heated debate over the ethical and psychological implications of this new type of relationship (Aulisio and Arora, 2014; Greenblum and Kasperbauer, 2018), the literature generally demonstrates its benefits in terms of its positive effects on the well-being of both healthcare professionals and patients (Fuertes et al., 2017). In some cases, the quality of patients' relationships with healthcare professionals seems to be even more important than active therapies targeting the disease, especially when there is little chance of recovery. Where chronic diseases are concerned, active care and the quality of relationships seem to be equal in importance (Hanganu et al., 2019). In recent years, several models have been developed. These focus on the development of healthcare professionals' relational/communicative skills (Dinkel et al., 2016; Adamson et al., 2019; Testoni et al., 2019b); shared responsibility in decision-making processes regarding the treatment plan; motivation (Kos, 2019; Lipovetski and Cojocaru, 2019); and trust (Hoff and Collinson, 2016; Ruberton et al., 2016; Chandra et al., 2018; Orom et al., 2018). However, as with any kind of human relationship, difficulties are inevitable—especially when doctors must make decisions that are painful for the patient (Restivo et al., 2018). In such cases, the empathy that physicians and nurses experience may cause them severe psychoemotional stress and heighten their risk of burnout (Zamperini et al., 2015). Healthcare professionals unconsciously deploy psychological defences aimed to distance patients from decision-making processes (Capozza et al., 2015). However, a recent study on a group of non-healthcare personnel showed that such defences do not reduce burnout levels (Testoni et al., 2020a). This demonstrated the need to find better strategies (Testoni et al., 2019a) [e.g., by enhancing the spiritual dimension (Testoni et al., 2016; Castro et al., 2019)]. This could be particularly helpful given the growing impact of new medical technologies on the healthcare provider-patient relationship, as such technologies often increase the distance between these parties (Pirone, 2018; Eyal et al., 2019; Dalton-Brown, 2020; Matthews, 2020; Sakka and Qarashay, 2020).

The COVID-19 crisis had serious consequences for public health and patients' medical care in Italy (Labrague and de Los Santos, 2020). It negatively impacted the healthcare provider-patient relationship as a result of the enormous surge in seriously ill, infected patients that overwhelmed the healthcare system (Ministry of Health, 2020; World Health Organization, 2020a). Physicians and nurses who had been trained to humanise their relationships with patients were suddenly forced to revert to older models of intervention. During the early phases of the pandemic, it was impossible for healthcare professionals to respond to patients' exigencies due to overcrowding in hospitals and insufficient staffing. Once can easily imagine the impacts of this peculiar psychological situation on mental health of healthcare professionals (Hossain et al., 2020a,b; Pfefferbaum and North, 2020). Similarly to what had occurred during the 2002–2004 severe acute respiratory syndrome (SARS) outbreak (Brooks et al., 2020), healthcare professionals were among the professionals who were most distressed (Labrague and Santos, 2020; Mo et al., 2020; Nemati et al., 2020), alongside patients (Guo et al., 2020).

Several psychological factors have been considered with regard to healthcare providers. First, according to recent studies (Jin et al., 2020; Sultana et al., 2020), the excessive number of working hours makes this group more susceptible to experiencing anxiety, depression, burnout, and insomnia. Additionally, healthcare providers feared contracting the coronavirus or infecting loved ones (Cao et al., 2020). In one study by Halcomb et al. (2020), this concern involved 80.9% of this worker population. Among the factors causing a high degree of stress was the reported fear of infection due to lacking or inadequate personal protective equipment (Chirico et al., 2020; Halcomb et al., 2020). Moreover, healthcare providers experienced high levels of stress in their attempts to mediate patients' needs compared with their own personal and family needs (Greenberg et al., 2020). Doctors and nurses were forced to make ethical decisions, taking into account the exceeded capacity of hospital wards and COVID-19 patients' medical needs (Xiang et al., 2020). In addition to the exacerbation of anxiety, depression and post-traumatic stress disorder (PTSD) (Alharbi et al., 2020), a 40% increase in compassion fatigue was noted (Van Mol et al., 2015). Indeed, physical and psychological stress can be related to difficulties in mediating personal needs in connexion with constant and prolonged demand for patient care (Ruiz-Fernández et al., 2020). This situation has led to social dislocation and traumatic experiences that have been demonstrated to carry the risk of gradual desensitisation and loss of compassion due to excessive exposure to suffering (Joinson, 1992). The inadequacy and the insufficiency of resources for protecting frontline healthcare providers' physical and psychological well-being also needs to be considered.

In light of these difficult circumstances, the purpose of our study was to examine narratives describing the experiences of physicians and nurses who worked in hospitals in two of the Northern Italian cities hardest struck by COVID-19. The most significant critical incidents involving hospital professionals and the related psychoemotional stress were analysed to understand what substantial changes could be made to the current healthcare system to improve care for both patients and their healthcare providers facing a similar future crisis. Critical incidents, as job-related stressors, affect the individuals involved both at the time of their occurrence and even years after the incident had passed. Exposure to critical incidents (such as the current response to COVID-19) is a particular concern in the field of healthcare quality, considering that such exposure has the potential to increase the already heavy workloads of physicians and nurses (Caldas et al., 2020). In Italy, guidelines were released to provide all healthcare professionals involved in the COVID-19 crisis with psychological support services centred on coping strategies for managing stress and anxiety (Chirico et al., 2020).

## Materials and Methods

### Objectives

This study utilised a qualitative research design method to investigate professional and psychoemotional stress among physicians and nurses during the COVID-19 pandemic, with a particular emphasis on their relationships with patients. We sought to understand if and how empathetic attention to patients and its humanising effect on medical care helped physicians and nurses cope with the pandemic. In particular, we were interested in the ways in which healthcare providers experienced the state of emergency imposed by the rapid spread of the coronavirus and the major difficulties they encountered. We paid special attention to the possibility that the critical incidents in which doctors and nurses found themselves during the worst period of the crisis (March to May 2020) might have generated significant psycho-emotional stress that undermined their empathetic relationships with their patients. A final aim was to identify, in participants' opinions, substantial changes and interventions that are deemed necessary to the Italian national health service.

### Participants

Our study examined healthcare professionals working in hospitals in two areas of Northern Italy most affected by the virus during the first phase of the COVID-19 pandemic. A total of 17 healthcare professionals from two hospitals in Northern Italy were enrolled in the study. Of these, six were nurses (five female and one male) and 11 were physicians (seven male and four female). The mean age was 47 years (SD = 9, range: 35–60 years). Their years of service ranged from 6 to 30 years (mean = 19.88, SD = 10.86) (Tables 1, 2). Three of the physicians who were interviewed worked in an intensive care unit and the remaining healthcare providers worked in general internal medicine departments, cardiology departments and infectious disease units. Because of the reorganisation of the hospitals in view of the pandemic, all the participants worked in close contact with COVID-19 patients. Recruitment began with a pre-research collaboration between the research team and two physicians. A discussion about the difficulties that healthcare professionals were experiencing motivated everyone to initiate this study. The two physicians, in turn, engaged physicians and nurses who felt able to talk about their experiences without it being too traumatic for them. The research team tried as much as possible not to further stress the participating healthcare providers, and the latter's ability to discuss their experiences was one of the selection criteria. Those who had experienced major psychological distress were already in treatment prior to the interview, and all participants could receive special counselling services.

**Table 1:** Participants' characteristics.

**Number of participants**		**Gender**		**Age**		**Length of service (in years)**	
**Physicians**	**Nurses**	**Male**	**Female**	**Mean**	**SD**	**Mean**	**SD**
11	6	8	9	47	9	19.88	10.86
(64.7%)	(35.3%)	(47.05%)	(52.94%)				

**Table 2:** Participants: physicians and nurses.

**Pseudonyms**	**Age**	**Length of service (in years)**	**Profession**
Alfredo	36	8	Physician
Amalia	52	21	Physician
Arianna	54	30	Nurse
Aurora	37	8	Physician
Camillo	40	9	Physician
Carlotta	35	8	Physician
Eleonora	58	30	Nurse
Lelio	55	30	Physician
Martina	56	27	Nurse
Matilda	35	7	Physician
Salvo	36	4	Physician
Salvatore	44	20	Physician
Serenella	36	13	Nurse
Sonia	50	25	Nurse
Tarcisio	53	33	Nurse
Valerio	57	31	Physician
Vittorio	60	34	Physician

### Data Collection and Analysis

The snowball sampling method was employed for recruiting participants. This non-randomised method is often used in qualitative research in healthcare disciplines because it is viewed as appropriate, especially when the members of a particular population are difficult to locate (Rubin and Babbie, 2010). In this study, each participant referred colleagues, both doctors and nurses, as potential participants. Participant recruitment was halted only when reported themes from the interviewed professionals became repetitive and the data achieved theoretical saturation. Two specially-trained psychological interviewers conducted the interviews, and an experienced psychologist and professor supervised these interviewers continuously. Although, considering that their colleagues had invited them, the participants were familiar with the objectives of the interview, the conception of the study and all its objectives were explained to the participants by the researchers. The participants were asked whether they felt comfortable enough to support the research and were required to sign an informed consent form before proceeding. They were also asked to confirm their consent after the interview. Individual semi-structured interviews were conducted through Skype, with a mean duration of 60 min per interview (SD = 15').

In line with principles and processes common to thematic analysis (TA) (Braun and Clarke, 2006), the study followed a qualitative research-in-psychology design that utilised in-depth interviews concerned with existential, personal and professional dimensions (Camic et al., 2003). The researchers' primary concern was to collect data from participants on the issues that characterise the phenomenon under investigation while considering three predominant factors: changes and problematic aspects after the beginning of the outbreak; relationships with patients during a state of emergency; and emotions and reactions related to COVID-19. Because the study aimed to make sense of how doctors think through their lived experiences and focused on their reactions and on how their work changed during the pandemic, the semi-structured interviews were inspired by Interpretative Phenomenological Analysis (IPA) (Smith and Osborn, 2008; Pietkiewicz and Smith, 2014). Allowing participants to express themselves freely, the dialogues were realised through computer-mediated communication, recorded and transcribed verbatim. The interviews aimed to elicit respondents' horizons of meaning, as is characteristic of IPA, and the analysis attempted to recognise the main themes that were common *among* interviewees rather than within each of them (Larkin et al., 2006). Similarly, as in other studies that integrate two different methodologies (in this case, TA and IPA) (i.e., Danivas et al., 2016; Thompson, 2017; Ferguson and McAuley, 2019), the texts underwent an analysis that aimed to identify similarities and specificities *across* all the narratives (Braun and Clarke, 2020). In this process, thematic patterns were identified using Atlas.ti software (Muhr, 1991). Common patterns and emergent themes were identified to illustrate convergences and specificities among all participants' answers through a systematic comparison across the texts. The connexions were identified and interpreted through abstraction, which allowed the researchers to recognise the main emergent themes (Leo and Goodwin, 2016; Rotenberg et al., 2020). Two of the authors jointly developed a temporary codebook using the transcripts and attempted to ground each code in the participants' narrated experiences. Together, in an iterative process, they extracted codes and identified sentences that contained a single theme. After this, the coding was organised to refine and reduce the various themes to produce inclusive main themes. All differences of opinion were resolved through discussion until the codes were agreed upon unanimously. The codes were assigned descriptive labels that were consolidated into themes, then reviewed and revised several times through discussion. Finally, a consensus was reached with additional, supervising authors. The flexibility of this approach allowed for unexpected issues to emerge from the narratives without the use of a structured hypothesis guided by the literature (Testoni et al., 2018, 2020c; Rotenberg et al., 2020). The analysis performed by the interviewer and supervisor followed six fundamental phases: preparatory organisation; generating categories or themes; coding data; testing emerging understanding; searching for alternative explanations; and writing up the report (Marshall and Rossman, 1999; Braun and Clarke, 2006; Testoni et al., 2020b). To verify the correctness of the analysis and interpretive procedures adopted by the interviewer and the supervisor, two other members of the research team worked on the texts until agreement was reached among all the researchers.

The study respected the American Psychological Association's Ethical Principles and Code of Conduct, as well as the principles of the Declaration of Helsinki. Furthermore, the study was approved by the Ethics Committee of the University of Padua (n. 8DD829A1F8F83852FEDB64AAE38A4F79). Participants were informed about the study's aims and procedures, and they were assured that participation was voluntary and that their responses would remain anonymous. Informed consent was obtained from all participants. In order to protect the participants' identities, the names used in this text are pseudonyms.

## Results

Analysis of the interviews identified the following fundamental themes.

### Disorganisation and Psychoemotional Stress

COVID-19's rapid spread exposed healthcare professionals to a completely unexpected and unpredictable scenario that caused intense psychoemotional stress, directly involving their relationships with patients. Arianna, a 54-year-old nurse, described her great difficulty in managing a situation for which she felt totally unprepared:

I had to deal with a flood of sick people, and I couldn't handle all the demands. Not only there was a lack of medical equipment, such as ventilators and masks, but I could not even respond verbally to patients' cries for help. I could not even go to the bed(s) of those who called me because they were choking to death. It was impossible to cure them. One simply tried to survive hoping that someone would be saved.

Almost all the participants cited this issue, and their descriptions of psychoemotional stress were associated with discouragement, helplessness and inadequacy, as exemplified by Eleonora, a 58-year-old nurse:

What scared me the most was to realise that the antipyretic, antibiotic and oxygen had no effect. Previously, we were used to seeing improvement in even the most serious patients, thanks to these interventions. Instead, this time, we didn't have enough drugs and controls, and they didn't have the effect we expected. It was frightening, and the experience of helplessness was terrible. All this made my relationship with patients very uncertain because I did not know how to reassure them.

This feeling of impotence was associated with a loss of hope, along with a profound sense of inadequacy caused by the repeated ineffectiveness of the instruments that were normally used. In other situations, these instruments were effective and would usually give these professionals a sense of validation. Tarcisio, a 53-year-old nurse, added: “We didn't have enough masks or gowns. The growing urgency had to deal with the expectations of the boxes that carried the protective material, which often did not fit, and we had to adapt to what was available.” Martina, a 56-year-old nurse, described a similar experience:

It was shocking to see that all our efforts were not having any effect on patients! Everything was useless because whatever we did, the patients died suddenly. Any effort, any administration of powerful drugs was like giving fresh water. No therapy had any positive effect. Then we started using palliative drugs, at least to soothe their suffering and make their agony less excruciating! There was nothing else we could do.

This sense of powerlessness and the associated distress were exacerbated by a latent form of resentment, an anger without a precise object. The most concrete object they could hold responsible was the regional healthcare system, which, being an impersonal entity, could be taken as a general background on which to project their feelings. Anger and resentment were accompanied by demoralisation and helplessness, as described by Serenella, a 36-year-old nurse:

About a 100 colleagues were taken from wards and clinics and moved to COVID areas to cope with the emergency. We came to this because there was a lack of personnel due to regional cuts in recent years. We were (used) as patches everywhere and were used as a stopgap here and there without anyone having the necessary expertise to deal with this emergency. The errors in the management of the health service have been a joint cause of this slaughterhouse. I hope that this terrible experience will make it clear that healthcare professionals are not a cost, but a resource for the true well-being of the population.

Psychoemotional stress had inevitable post-traumatic psychosomatic repercussions. Lelio, a doctor in internal medicine, reported:

I relive continuously, day and night, the experiences lived during the first period of the pandemic—the urgency, the ineffectiveness of drugs, the deaths. Even now, it is as if I continue to work even when I am asleep because problematic situations reoccur, and sometimes the possible solutions I could have adopted come to mind. I keep thinking about it because I always want to give my best, and flashbacks require me to rethink what can be done to solve this situation.

The expression of a need for psycho-emotional distancing came through computer-mediated communication. This strategy was adopted because of thepsychological need to safeguard both physical health and psychological well-being. In fact, patients understood that physicians did not want to see them in person and, therefore, did not object. This simplification helped family physicians avoid becoming too emotionally involved through face-to-face relationships with patients.

Many healthcare professionals worked on wards where they did not normally deal with contagious diseases, yet were summoned to serve in COVID emergency wards and assigned tasks beyond their training, expertise and experience, as witnessed by Tarcisio:

It was all sudden and suddenly unmanageable. I found myself literally catapulted from my department to the COVID one without any preparation. We all found ourselves working without knowing anything about each other, only names written on suits so that we could at least call each other by name without making mistakes. No training in the use of the new protocols, all improvised and all to learn from time to time, quickly, without time to compare ourselves with each other. And as soon as we learned something, we had to change because something new changed the whole system of functioning of relationships and treatments.

With respect to the problems they were confronted with and the readiness required of them in this situation, all participants expressed the need for specific training to be prepared for handling similar emergency working conditions in the future. All participants felt that they had been unprepared for the COVID-19 challenge. No one was equipped to handle the sudden escalation in deaths and ineffective care. Their idea was that a specific education on emergency and pandemic could have prepared them to prevent trauma. As Tarcisio suggested:

COVID made us understand that in our healthcare system, in our organisation and in our training, there is a great deficiency: We have never considered the possibility that something exceptional, as a pandemic is, could happen. It is clear that this is a mistake that we can no longer make and, therefore, it is necessary to introduce special training courses and provide suitable protocols for these eventualities. It is necessary to do, like the firefighters who practise to always be ready.

### Urgency and Critical Incidents

Incidents in care relationships can lead to healthcare professionals wanting to quit their jobs. This phenomenon did not emerge among our participants, but rather came to light in their narratives concerning the emotional work that would not allow them to leave the field. They clearly described how the urgency of the situation prevented them from becoming immediately aware of their emotions, as nurse Martina described:

The work had become not only useless, but also frenetic. There was not enough time for anything, for thinking, for trying to find new solutions. There wasn't even time to cry or even to realise the pain we were feeling. It had to be done, it had to be done… and all in a hurry…. The number of sick people kept growing out of all proportion, and there wasn't time for everyone. However, the most frequent critical incidents were related to the fact that we did not arrive in time to prevent them from dying.

For Martina, the effect was damaging, and she still has visions of the traumatic scenes from the pandemic's first phases. Martina did not think about quitting her job or shirking her professional responsibilities, but she still had to withdraw from certain spaces:

In the hospital, in some rooms, I still see the traumatic scenes related to the explosion of the pandemic. Even now, when I am on duty, when I enter some room(s), I see patients who could not breathe, in bed, who died suddenly. I still have these flashes that still shock me, especially when I enter two rooms in particular […]. Whenever possible, I try to avoid going into those rooms so I don't relive those memories.

The feeling of not being able to count on the support and protection needed from the healthcare system; the desire not to give in to discouragement; and the determination not to abandon the field have led many of these professionals to seek personal solutions to safeguarding their health and continuing to help the sick responsibly. Matilda, a 35-year-old emergency medical doctor, declared: “They didn't even give us masks to defend ourselves. To avoid getting infected, I took courage and bought the masks on the internet and went to visit the sick, knowing that perhaps that tool might not be enough.” Similarly, nurse Eleonora added the following:

We didn't have the appropriate equipment to protect ourselves. The ones we had were scarce, and as (there was) not enough for everyone, we had to wear them all day or all night without ever giving them up, knowing that in that way, they were saturated with contaminants and viruses.

One of the most important dimensions thrown into crisis by the emergency situation was the relational dimension. Suddenly, all previous professional training in relating humanely and empathetically with patients became futile. Because of the pandemic, the healthcare provider-patient relationship abruptly became impersonal, and the sick could no longer be cared for as people. Their humanity had to be put on the back burner as a practical matter. Added to the accompanying sense of impotence, discouragement, and anger was the fear of a loss of humanity in the professionals' relationships with the sick. Lelio's trauma seemed to be caused by his empathy for his patients:

We were constantly on edge because we were dealing with extremely scared people. They felt isolated, even though they hadn't been abandoned by their loved ones, and I suffered seeing their anguish because they knew they couldn't see their family members again. We couldn't do anything. We worked in terror.

Underlying the despair, anger and terror was the ethical problem of managing the scarcity of life-saving devices (e.g., oxygen). Participants often found themselves having to choose whom to save and whom to let die—and to do so quickly. This was a stark contrast to what they had previously learned about respecting the rights of every patient as a person and thereby guaranteeing minimum levels of care regardless of age, gender, ethnicity, or status. Nurse Arianna's narrative continued in this vein:

It was traumatic because we were absolutely unprepared to manage such a situation and the relationship with patients in such a condition. We did not know how to move, we were not organised and we had to improvise in the constant emergency. No one expected this situation, and we were caught unprepared. We were constantly too late to save patients […] We would have liked to cure everyone, but it was impossible. The most difficult incidents to manage were those caused by lack of resources. I'm sorry to say, but that's how it happened. We had to make ethical choices: If a young person came in urgently needing care, we gave priority to him/her and left aside the older ones, who then died. And for us, it was traumatic because we had not been able to treat them.

The narrative of Salvatore, a 44-year-old resuscitation physician, was similarly dramatic, although it described a totally different perspective. While Arianna talked about sacrificing elderly people to use life-saving medical devices on younger people, Salvatore found himself dealing with a tragic decision from the other end. A young mother was not treated as she could have been, which put him in deep crisis:

We had to manage a patient who was in her 40's and was very healthy. The only chance we had to save her from COVID was to stabilise her and give her a heart transplant. My department head asked his superior for permission, who denied us the option. We all collapsed in despondency because we were forced to obey. We were not permitted to proceed with this course of treatment, and we let this woman die. She had only that one chance of survival. Instead, we continued to treat the elderly with therapeutic obstinacy, even when there was nothing left to do. Instead, for this young woman with three young children at home who needed her, we could do nothing. This incident to me is completely unacceptable. It was a monstrous decision.

The loss of all protocols and balance made the work environment a minefield where nothing was predictable anymore, producing upheaval not only in the participants' professional lives, but their personal ones, as well. Aurora, a 37-year-old female general practitioner, testified to this:

All the work was an accident. Nothing worked the way it used to, and not only did (I) not recognise my job, but my whole life was turned upside down. […] I was so upset that for a while, I refused to answer the phone. I didn't want to hear from friends or relatives because I couldn't even speak because of the level of stress, anxiety and suffering. I even avoided my parents for fear of answering them badly after terrifying days. The anxiety was so strong that I couldn't get to sleep, even though I was so tired.

### Everything Surreal

A factor that further caused deep discomfort during the first phases of the pandemic was the change in the organisation of work and in the care and protection equipment to be adopted. These professionals were totally unprepared for this change, and one of the biggest difficulties was having to learn how to use new medical tools rapidly and without any previous training in a controlled environment. Amalia, a 52-year-old doctor, described the medical devices:

At first, we didn't even have the usual instruments to protect ourselves. Then came the ones you need to use to defend yourself from infection. Now we are dressed like divers in heavy wetsuits, which do not allow us to drink, eat or go to the bathroom normally. We can't sit for 10 h in a row, and we arrive at the end of the shift exhausted and eager only to wash and sleep, without thinking about anything else because we don't have the strength to think or do anything anymore.

Having to work intensely amid the hectic and chaotic unpredictability of events was experienced as immersion in an uninhabitable space. Diverse terms were used to describe the protective medical suits. Lelio, like Amalia, called it a “diving suit,” while Valerio, an internist and colleague of Lelio's, referred to it as a “sarcophagus.” The most intensely stressful psychoemotional effect was that of finding oneself in a condition of sensory deprivation, as though in a coffin. Furthermore, movements were extremely difficult to perform, as Amalia described:

Since the material was not always adequate, we found ourselves working with three pairs of gloves and huge, bulky, heavy suits that were not even our size, so each movement was awkward, slowed, braked, made impossible. The visor also fogged up, and it was difficult to see.

The shared feeling was that of being constantly immersed in an unreal situation. Lelio, who suffered from his empathy for patients, expressed his discomfort in these terms:

Dressed like that, we appeared to the patients as Martians because we looked like astronauts, or divers, completely hidden by the diving suit. We were no longer recognisable as people, and the only thing that indicated who we were was our name because we wrote it on our suits with a marker pen so that the patients could recognise us. It was all surreal, like being suddenly thrown into space.

Not all strategies for coping with emotional distress were equally effective for everyone. For Tarcisio, writing one's name on the suit was not enough:

I didn't recognise people, and I couldn't always distinguish if those I had in front of me were doctors or nurses. The whole team had changed because many professionals came from other departments, so even the relationship between us colleagues could not be based on previously established mutual knowledge.

Valerio experienced the reduction in physical contact with patients as a substantial loss: “The suits were like uniforms; they made us unrecognisable, all the same. We no longer understood who was a man and who a woman, and at the same time, we no longer perceived reality as we normally do […]. This form of relationship is really stressful.”

In addition to the difficulties linked to protective and preventive measures, the reorganisation of the hospitals' departments was a source of stress. Because of this reorganisation, everything that had previously been learned seemed to lose consistency and usefulness, while improvisation and rapid adaptability at any cost seemed to be the only measures to which anyone—independently—could resort. The loss of all referent launched doctors and nurses into a frenetic and chaotic space deprived of meaning related to their professional and human experiences.

### Disruption of the Empathetic Relationship With the Patient

The loss of all human, ethical and deontological reference point concerning their relationships with patients rattled almost all participants. If this was the most critical underlying aspect of pandemic management, it was no less difficult for the participants to have to modify every aspect of their relationships with patients without any prior expertise on alternative models. Indeed, the state of emergency completely undermined their relationship with patients, and uncertainty contributed to the loss of all empathetic capacity. The uncertainty caused by the lack of effective drugs, medical equipment, and adequate time for patients forced participants to have relationships characterised by great insecurity. The core values that once guaranteed caring relationships suddenly became “pretty words” that had nothing to do with reality. Tarcisio expressed this idea in a strongly dismayed tone that also conveyed harsh disillusionment:

There is much talk about ethical issues related to the relationship with the patient and informed consent. I work in the emergency room, and there, it was absolutely impossible to negotiate any kind of decision with patients. Here, patients just do what they are told. There is no room to negotiate treatment because there is no time. During the pandemic breakdown, we also did what was possible and always late in our response due to the absolute lack of time. We could never explain anything because we did not know how to handle the urgency either. I experienced a lot of stress because I wondered if it was right that they were subjected to our decisions without knowing what was waiting for them, while we knew that everything we were doing could be useless.

The total psychological distancing from patients resulted in the participants' perception that they were dealing not so much with human beings, but, rather, with things akin to furniture. This perception of the dehumanisation of relationships was expressed clearly by Salvatore:

In resuscitation, we sedated all the patients and intubated them. It was impossible to have a relationship with them, and, little by little, we got used to this kind of relationship, so much so that in the end, we saw them as part of the furnishings. So, it is certainly easier to treat the patient.

Another form of distancing was conducted particularly by family physicians through the use of phone- and computer-mediated communication. As noted earlier, it was understandable to patients that their physicians would want to maintain physical distance so as not to become infected. However, this, in fact, also allowed physicians to distance themselves from the psychoemotional distress that in-person contact implied. Alfredo, alluding to the theme of the shared planning of care with patients, said:

We were trying hard, and we tried to make the patients understand it too. They also understood that we could not do more and they became less argumentative. Many patients then contacted me *via* email to encourage me and to thank me. I used internet counselling a lot, and this allowed me to keep my distance, and the patients also understood that it was easier that way […] This helped me to better manage their requests without being overwhelmed by their anxiety.

Amalia further emphasised patients' greater willingness to cooperate, pointing out that the pandemic had deconstructed the classic script in which patients are always ready to clash with physicians:

They were also scared and maybe even disoriented, so they were less polemic(al) than what our work was. We all felt that even the patients understood that we were in trouble and that we were trying hard. The patients understood that none of us was prepared to death with this.

Distancing was also practised through protective equipment that made any empathetic contact even more difficult. Indeed, healthcare professionals' ability to express closeness to and empathy with the sick, even as they neared death, was severely hampered. Lelio described this difficulty in relating to patients as follows:

The suit has certainly reduced the anxiety of coming into contact with sick patients. Before, we lived in terror. When we were able to wear these new medical devices, we felt personally safer, but even further away from the patients. The suit creates an almost insurmountable distance; it is a real barrier between the doctor and the patient. The empathetic relationship becomes absolutely impossible, and even on a perceptual level, the chances of coming into contact with patients (were) reduced. As much as we wanted to establish a dialogue, in fact, even the words were suffocated by the masks. No facial expressions could support communication. The gestures were awkward, and no physical contact was possible except those necessary for the treatment. Honestly, it was very difficult to go beyond formal gestures. However, the patients seemed to understand that we were in trouble, and they seemed very careful not to put us in any further difficulty. Both they and we were trying to adapt to each other's needs.

The need for distancing is a response to severe distress. The greater the importance previously placed on the empathetic relationship with the patient, the greater the anxiety over contagion. Martina suffered greatly through this situation:

After all we did, they died badly in the end. The doctors could make every attempt possible, but they couldn't save them. They died with a hunger for air, and it was terrible to see them die like that […] I have always given a lot of importance to the relationship with the patients. One night, however, I was assailed by the anxiety of contagion, and a seriously ill patient was asking for me insistently. I knew that she was dying and that I had to be close to her, holding her hand to make her feel my closeness and accompany her through the passage. I literally escaped from that room. The patient died. I will always remember her because she kept calling us and telling us not to “leave me here alone—don't leave me here alone” because she felt she was dying, but being inside meant you get sick too.

The perception of being faced with a situation in which the patient-centred model of care and the empathetic relationship were being challenged was a further cause of disorientation and emotional distress. Eleonora highlighted the discrepancy between her patient-centred approach training and its lack of applicability during the COVID-19 crisis, noting the need to find strategies for applying the same model in extreme contexts—particularly in patients' final stages of life:

Patients were left to their own devices, and everything we usually do to enhance the care relationship had failed. Usually, when you accompany a terminal patient to death, you call the minister of worship, who helps the dying person get in touch with their spirituality. Usually, we hold his hand so that he does not feel alone in the passage. At this juncture, instead, we only wait for him to die to close him in a black bag and seal him well because this is the procedure to be used. As soon as the patients die, you close them in the black bag and take them to the mortuary to wait for the funeral home to pick him up. There is no more of those empathic relational modalities that characterise the end of life of other patients. The operations are now aseptic, really cold. I am not used to working like this because I give a lot of importance to the empathic relationship. I can't stand to treat patients (like) numbers. I can't stand the lack of a deep relationship with those who die.

Aurora described the same experience:

I was used to having a very empathic relationship with my patients in the nursing home. I was accustomed to the caress of kissing, so a touch on the arm on the shoulder. My patients really need to feel my closeness in this way too. These things are impossible now, and the patients don't realise this because they don't understand what is happening. So, they still try to get their hands close to my face; they are somehow looking for closeness. It's all really difficult to manage now.

The increasingly widespread promotion of the palliative care model in Italy over the last decade has prepared most physicians and nurses to take patients' conditions into huge consideration, even during the terminal phase of their illness. Therefore, it has been very difficult for them to manage patients' deaths so coldly. Perhaps one of the worst moments for all participants was related to the management of corpses, as Martina noted:

The most dramatic moment was when we had to learn how to manage the black bag. We had to learn to immediately close the corpses in the black sacks to have them taken immediately to the mortuary without blessing, without anyone having said goodbye. We had to store them in the mortuary and leave them there. Put them in the archives to wait their turn to leave and be cremated or buried quickly by the funeral home. And then close everything with papers, documentation and administrative practises. No, I was not ready to face all that.

**Figure 1:**
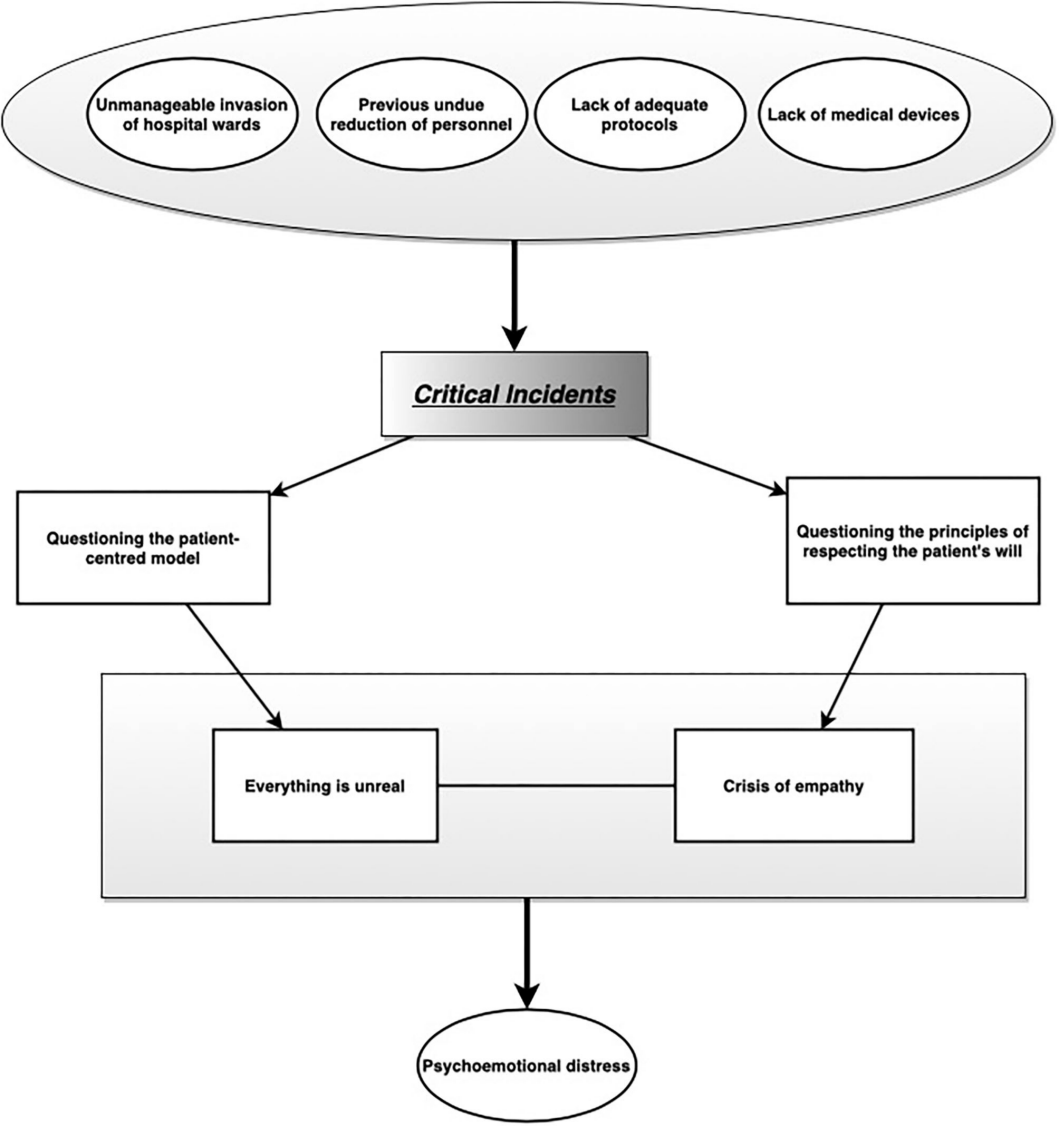
Findings of qualitative data analysis. Main themes and their relationships.

Figure 1 summarises the major components of the interviews' most salient themes' by highlighting the relationships among them.

## Discussion

This study examined healthcare workers' lived experiences during the COVID-19 pandemic. Previous research had demonstrated that healthcare workers experienced significant psychoemotional stress during the SARS and MERS epidemics (Tam et al., 2004; Lee et al., 2018). Our study found that inadequate emergency preparation and limited resources in the Italian healthcare system elicited disastrous results after the unexpected arrival of COVID. Four closely-linked, main themes demonstrated the distressing effects on healthcare professionals of such lack of preparation: (1) disorganisation and psychoemotional stress; (2) urgency and critical incidents; (3) everything surreal; and (4) disruption of the empathetic relationship with patients.

The shortage of operational protocols and the lack of personal protective equipment needed for the crisis have been thoroughly documented worldwide (Livingston et al., 2020; Ranney et al., 2020). Based on the World Health Organization's (2020b) expectations, an estimated 89 million medical masks, 76 million examination gloves, and 1.6 million goggles are needed for the COVID-19 response each month. In Italy, as evidenced by Arianna's narrative, limited access to medical equipment such as medical masks and respirators left frontline healthcare workers ill-equipped to care for COVID-19 patients. This lack of personal protective equipment has been associated with increased psychoemotional stress among healthcare workers, thereby hindering their ability to cope with the crisis (Fernandez et al., 2020). Our results align with what other scholars have already demonstrated; healthcare professionals had higher perceived anxiety, insomnia, and overall psychological problems (Barello et al., 2020; Que et al., 2020). In our participants' narratives, the psychological and physical impacts of the state of emergency have been described mainly as difficulty falling asleep and having ruminative thoughts. A systematic review and meta-analysis developed by Pappa et al. (2020) provided early evidence that a high proportion of healthcare professionals experienced mood and sleep disturbances during the COVID-19 pandemic. As confirmed by Eleonora's and Martina's interviews, healthcare professionals experienced helplessness and frustration with regards to patients' suffering and sudden death.

Studies of past epidemics have shown that frontline healthcare professionals were at higher risk of mental health disorders, such as post-traumatic stress disorder, after the epidemic (Liu et al., 2020). Our research highlights additional difficulties. The lack of available treatments and protocols to guide both clinical management and prioritisation in caring for COVID patients was a source of suffering and distress. In particular, we refer to the narrative of Salvatore, who witnessed a young mother die without being able to do anything for her while elderly patients were given priority, with extraordinary effort directed towards treating them. His situation contrasted with Arianna's; due to a lack of resources, Arianna and her colleagues chose to save the youngest at the cost of the oldest. Both Salvatore and Arianna suffered not only from the ethical dilemmas that they faced without adequate preparation and guidance, but also from the need to alter their relationships with their patients. The first theme from these critical incidents (i.e., disorganisation and psychoemotional stress) punctuated the narratives of all participants, who began their accounts by highlighting the shock they suffered when finding themselves unprepared to handle the crisis without proper tools and protective equipment. As seen in the second theme (urgency and critical incidents), they then remarked on the importance of the relationship with the patient and how this was jeopardised by the lack of tools and intervention models. According to Tarcisio's narrative, the pre-existing shortage of healthcare providers forced the sudden redeployment of medical professionals in order to optimise resources for the pandemic. This was linked to the perception that everything was unmanageable due to substantial disorganisation within the healthcare system, which rendered all relationships unrecognisable and meaningless. In Tarcisio's opinion, even the principle of informed consent had been transformed into a rhetorical issue without any operational value. Throughout the emergency, all participants tried their best to provide psychological support for their patients, but exhaustion, fear of contagion and use of protective equipment made it impossible to establish empathetic relationships. The COVID-19 pandemic has threatened the cornerstones of modern patient-centred medicine; the sheer volume of seriously ill patients and the lack of healthcare providers and resources have limited interaction time with patients, especially in emergency care. Thus, psychoemotional stress sprang from the reality that participants could not use their training in humanising the provider-patient relationship while caring for COVID patients.

Indeed, one of the most important issues, in our opinion, relates to the fact that all participants were strongly aligned with the patient-centred approach and, therefore, were accustomed to exercising an empathetic relationship with patients. Almost all reported work-related challenges, such as the safe delivery of care to patients while wearing personal protective equipment for several hours. The presence of physical distress due to safety measures was consistent with the literature (Liu et al., 2020). The huge staffing shortages have been a major concern that influenced healthcare providers' ability to cope with the demanding workload during the pandemic (Fernandez et al., 2020). Time devoted to better understanding each patient's expectations, feelings and fears—which lies at the foundation of a patient-based approach (Levenstein et al., 1986)—is impossible during a pandemic because time is prioritised for triage and treatment. This problem is closely linked to the psychoemotional stress caused by the serious ethical dilemmas healthcare professionals have faced.

Given our findings, we believe that a combination of adequate training and psychological support for hospital healthcare workers is important when disasters strike. This is particularly true in the instance of infectious disease pandemics, as has been evidenced by past pandemics. In fact, we believe that doctors and nurses should be prepared to modulate their empathy and closeness to patients by knowing how to regulate relational availability on the basis of concrete situations—be these normal or exceptional ones. It is necessary to define precise protocols on how to guarantee the best type of relationship with the patient from psychological and ethical perspectives. Of course, it is impossible to establish from this preliminary investigation how these protocols should be set up. Further research is required in this regard.

## Conclusion

Through our analysis of the narratives obtained through in-depth interviews, we identified several important issues that must be taken into account in future educational and professional training for healthcare professionals. In particular, we found that the COVID-19 pandemic subverted the symbolic referents that normally surround the humanisation of care in medicine. It did so by undermining healthcare professionals' basic ethical protocols; the professionals found themselves unable to comply with these protocols without having alternative reference models. In our participants' opinions, systematic and in-depth psychological training is needed to prepare them for crisis conditions in terms of their altered relationships with patients; the use of exceptional equipment; the preparation of new bioethical models suitable for disasters and pandemics; and engagement with the themes of death and dying. Indeed, it is important to prepare healthcare professionals so that the profound feelings of discouragement that can grow out of exceptional situations such as COVID-19 can be avoided during similar states of emergency.

## Limitations and Future Research Directions

This study has some limitations, particularly concerning the impossibility of generalising the results, but it provides a much-needed foundation for future research on healthcare workers' difficulties and needs in managing acute states of urgency, thereby filling a current literature gap. In fact, further studies should investigate further frontline workers' experiences following unpredictable events and focus on what kind of professional interventions could be particularly helpful in sustaining their concrete necessities. Therefore, it could be very useful to analyze the psychological outcomes of the COVID-19 pandemic in the healthcare worker population to develop a more adequate support system for these professionals in future crisis situations. We believe that a crucial node of the entire research is the ethical questions involved, considering that important discrepancies emerged: For some, too much precedence was given to the elderly, while others felt that the young should have been prioritised. This specific issue requires a very thorough investigation, and everything should be considered in relation to the ethical models adopted to regulate the decision-making process in such crisis situations while simultaneously addressing its potential impact on “emotional labour” and “moral distress.”

## Data Availability Statement

The raw data supporting the conclusions of this article will be made available by the authors, without undue reservation.

## Ethics Statement

The studies involving human participants were reviewed and approved by The Ethical Committee for the Psychological Research of the University of Padua. The patients/participants provided their written informed consent to participate in this study. Written informed consent was obtained from the individual(s) for the publication of any potentially identifiable images or data included in this article.

## Author Contributions

IT: project ideation, research design, supervision, analysis, article writing, and coordination. CF: article writing. EG: project ideation and interviews. EI: analysis, coordination, and supervision. CP: research design and supervision. RC: research design, supervision, and article writing. All authors contributed to the article and approved the submitted version.

## Conflict of Interest

The authors declare that the research was conducted in the absence of any commercial or financial relationships that could be construed as a potential conflict of interest.
